# Ca^2+^ Influx through Store-operated Calcium Channels Replenishes the Functional Phosphatidylinositol 4,5-Bisphosphate Pool Used by Cysteinyl Leukotriene Type I Receptors*

**DOI:** 10.1074/jbc.M115.678292

**Published:** 2015-10-14

**Authors:** Abdullah Alswied, Anant B. Parekh

**Affiliations:** From the Department of Physiology, Anatomy, and Genetics, University of Oxford, Oxford OX1 3PT, United Kingdom

**Keywords:** calcium channel, calcium intracellular release, calcium release-activated calcium channel protein 1 (ORAI1), phosphatidylinositol kinase (PI kinase), phosphatidylinositol signaling

## Abstract

Oscillations in cytoplasmic Ca^2+^ concentration are a universal mode of signaling following physiological levels of stimulation with agonists that engage the phospholipase C pathway. Sustained cytoplasmic Ca^2+^ oscillations require replenishment of the membrane phospholipid phosphatidylinositol 4,5-bisphosphate (PIP_2_), the source of the Ca^2+^-releasing second messenger inositol trisphosphate. Here we show that cytoplasmic Ca^2+^ oscillations induced by cysteinyl leukotriene type I receptor activation run down when cells are pretreated with Li^+^, an inhibitor of inositol monophosphatases that prevents PIP_2_ resynthesis. In Li^+^-treated cells, cytoplasmic Ca^2+^ signals evoked by an agonist were rescued by addition of exogenous inositol or phosphatidylinositol 4-phosphate (PI4P). Knockdown of the phosphatidylinositol 4-phosphate 5 (PIP5) kinases α and γ resulted in rapid loss of the intracellular Ca^2+^ oscillations and also prevented rescue by PI4P. Knockdown of talin1, a protein that helps regulate PIP5 kinases, accelerated rundown of cytoplasmic Ca^2+^ oscillations, and these could not be rescued by inositol or PI4P. In Li^+^-treated cells, recovery of the cytoplasmic Ca^2+^ oscillations in the presence of inositol or PI4P was suppressed when Ca^2+^ influx through store-operated Ca^2+^ channels was inhibited. After rundown of the Ca^2+^ signals following leukotriene receptor activation, stimulation of P2Y receptors evoked prominent inositol trisphosphate-dependent Ca^2+^ release. Therefore, leukotriene and P2Y receptors utilize distinct membrane PIP_2_ pools. Our findings show that store-operated Ca^2+^ entry is needed to sustain cytoplasmic Ca^2+^ signaling following leukotriene receptor activation both by refilling the Ca^2+^ stores and by helping to replenish the PIP_2_ pool accessible to leukotriene receptors, ostensibly through control of PIP5 kinase activity.

## Teaser

**Background:** Phosphatidylinositol 4,5-bisphosphate levels need to be replenished during calcium signaling, but how this is achieved is unclear.

**Results:** Ca^2+^ entry through Orai1 channels contributes to the resynthesis of phosphatidylinositol 4,5-bisphosphate and, thereby, helps sustain cytoplasmic Ca^2+^ oscillations.

**Conclusion:** Ca^2+^ influx sustains cytoplasmic Ca^2+^ oscillations by regulating phosphatidylinositol-4-phosphate 5-kinase.

**Significance:** Store-operated Ca^2+^ influx is important in maintaining phosphatidylinositol 4,5-bisphosphate levels.

## Introduction

A rise in intracellular Ca^2+^ concentration ([Ca^2+^]*_i_*) is a universal signal used throughout the phylogenetic tree to activate a broad range of spatially and temporally distinct cellular responses ranging from exocytosis and fast-twitch muscle contraction to nuclear gene expression and cell proliferation (1, 2). Inherent to the use of a multifarious signal like Ca^2+^ is the problem of specificity. How can one Ca^2+^-dependent response within a cell be activated but not another? Evidence is now accumulating that the size, time course, and subcellular location of the rise in [Ca^2+^]*_i_* are all important factors that contribute to the selective recruitment of downstream targets (2, 3).

In many cells types, stimulation of surface receptors that couple to heterotrimeric G_q_ proteins and phospholipase Cβ and, thereby, hydrolyze the membrane phospholipid PIP_2_^3^ generate the second messenger InsP_3_, which releases stored Ca^2+^ by opening ligand-gated Ca^2+^ channels on the endoplasmic reticulum (ER) (4). Low levels of stimulation of these G-protein-coupled receptors, considered to represent physiological levels of receptor activation, often result in the generation of repetitive oscillations in [Ca^2+^]*_i_* that present either as a series of baseline Ca^2+^ spikes or slower sinusoidal Ca^2+^ waves on an elevated plateau (5). Information is encoded in the amplitude, frequency, and spatial profile of the oscillation (3). Oscillations in [Ca^2+^]*_i_*, as opposed to a sustained rise in [Ca^2+^]*_i_*, enhance mitochondrial energy production (6), exocytosis (7), and Ca^2+^-dependent gene expression (8, 9) while avoiding the toxic effects that are associated with prolonged elevation of [Ca^2+^]*_i_*.

Oscillations in [Ca^2+^]*_i_* require either oscillations in the levels of InsP_3_ or biphasic gating of the InsP_3_ receptor by cytoplasmic Ca^2+^ in the presence of a steady increase in InsP_3_. Regardless of the mechanism, two conditions need to be satisfied for repetitive oscillations in [Ca^2+^]*_i_* to occur. First, the Ca^2+^ content of the stores must be maintained at a level sufficient for Ca^2+^ release. This is necessary because, following each Ca^2+^ release event, a fraction of the mobilized Ca^2+^ is extruded from the cell by plasma membrane Ca^2+^ATPase pumps (10). In the absence of refilling, store Ca^2+^ content would therefore drop below the level that supports InsP_3_-dependent Ca^2+^ release. Store refilling is accomplished through activation of store-operated CRAC channels in the plasma membrane that open following loss of Ca^2+^ from within the ER (11). CRAC channels are comprised of STIM and Orai proteins (12). STIM1 and STIM2 are ER Ca^2+^ sensors and migrate toward the plasma membrane upon store depletion (reviewed in Ref. 13). Orai1 is the pore-forming subunit of the CRAC channel and is gated by STIM binding to the C and N termini of the protein (reviewed in Ref. 14). The second criterion that needs to be satisfied for the generation of prolonged oscillations in [Ca^2+^]*_i_* is that PIP_2_ levels must be replenished following each Ca^2+^ spike to support production of the InsP_3_ that is needed for the next oscillation in [Ca^2+^]*_i_*. InsP_3_ has a lifetime of just a few seconds in the cytoplasm (15, 16) because of the presence of strong catabolic pathways. These breakdown pathways are well characterized and involve sequential dephosphorylation by phosphatases to myoinositol monophosphate, which, in turn, is dephosphorylated to myoinositol by inositol monophosphatases (IMPases), enzymes that are inhibited by Li^+^. Inositol combines with cytidine diphosphate diacylglycerol to form phosphatidylinositol (PI), which can then be phosphorylated by PI4 and PIP5 kinases to make PIP_2_.

Two IMPase genes have been identified in mammals: IMPase1 and IMPase2. IMPase2 is implicated in the pathogenesis of bipolar disorder, schizophrenia, and febrile seizures (17). Blocking of inositol monophosphatases, which would deplete PIP_2_ levels and, therefore, impair production of InsP_3_, is a possible mechanism to explain the mood-stabilizing effect of Li^+^ treatment on bipolar disorder (18). Although both IMPases are inhibited by Li^+^, the IMPase1 is more sensitive to blocking, with an IC_50_ of 0.7 mm (in 2 mm MgCl_2_) that is ∼30-fold left-shifted compared with IMPase2 inhibition (19).

PIP_2_ levels regulate CRAC channel activity in various ways. STIM1 trafficking to the cell periphery is expedited by the presence of a cytoplasmic Lys-rich domain that may bind to PIP_2_ and phosphatidylinositol 3,4,5-trisphosphate in the inner leaflet of the plasma membrane (20, 21). Furthermore, slow Ca^2+^-dependent inactivation of the channels requires Orai1 confinement to PIP_2_-rich domains of the plasma membrane (22). Here we explored the possibility that Ca^2+^ influx through CRAC channels, in addition to refilling the stores, regulates replenishment of the agonist-sensitive PIP_2_ pool during oscillations in [Ca^2+^]*_i_*. We find that the step converting PI4P to PIP_2_ is dependent on Ca^2+^ entry through the channels, providing an autoregulatory mechanism through which CRAC channels maintain their own activity by ensuring sufficient PIP_2_ levels in the plasma membrane for store depletion via InsP_3_ production.

## Experimental Procedures

### 

#### 

##### Reagents

Culture medium and salts were obtained from Sigma. LTC_4_ was purchased from Cambridge Bioscience Ltd. (Cambridge, UK). FCS, Fura-2/AM, Dulbecco's modified Eagle's medium, and penicillin-streptomycin were obtained from Invitrogen. Thapsigargin was purchased from Merck Chemicals Ltd. (Darmstadt, Germany). PI4P diC8 and unlabeled shuttle PIP carrier 3 were purchased from Echelon Biosciences Inc. Unlabeled shuttle PIP carrier 3 (catalog no. P-9C3) was used to deliver PI4P into cells. The carrier was reconstituted in distilled water and mixed with PI4P. The carrier-PI4P complex was vortexed and left at room temperature for 15–20 min. Following this, the complex was diluted in external solution and applied to intact cells for 7 min prior to stimulation with LTC_4_. The carrier final concentration was 20 μm.

##### Cell Culture

Rat basophilic leukemia RBL-2H3 cells were from the ATCC. Early experiments to see whether LTC_4_ evoked Ca^2+^ signals in this cell line were carried out on cells provided by Dr. Shamshad Cockcroft (University College London, London, UK). RBL-2H3 cells were used because of their strong secretory response, which is being investigated in a broader study within the group. Cells were routinely maintained in Dulbecco's modified Eagle's medium and supplemented with 10% FCS and 1% penicillin-streptomycin, as described previously (23). Cells were kept in an atmosphere containing 5% CO_2_ and maintained at 37 °C. For calcium imaging experiments, cells were plated onto 13-mm glass coverslips using 0.05% trypsin and used 48 h after passaging.

##### Cytoplasmic Ca^2+^ Measurements

A Nikon Eclipse TE2000-U inverted microscope equipped with an IMAGO charge-coupled device camera-based system from TILL Photonics (Gräfelfing, Germany) was used as described previously (23). Cells were alternately excited at 356/380 nm with exposure times of 20 ms and an acquisition rate of 0.5 Hz. Data were analyzed after import to IGOR Pro (Wave Metrics, Lake Oswego, OR). All experiments were performed at room temperature, and cells were kept in the dark when loaded. For cytoplasmic Ca^2+^ measurements, cells were loaded with Fura-2/AM (4 μm) for 40 min in an external solution comprised of the following: 145 mm NaCl, 2.8 mm KCl, 2 mm CaCl_2_, 2 mm MgCl_2_, 10 mm d-glucose, and 10 mm HEPES (pH 7.4 with NaOH). Calcium-free solution was of a similar composition as the external solution, except that 0.1 mm ethylene glycol-bis-(β-aminoethylether)-*N*,*N*,*N*′,*N*′-tetraacetic acid was substituted for calcium chloride. The number of calcium oscillations was quantified using IGOR Pro. Ca^2+^ spikes were considered to be oscillations when the R_peak_ − R_base_ value was >0.1.

##### Li^+^ Treatment

LiCl dissolved in DMEM replaced normal DMEM on cultured cells growing on coverslips. Cells were then maintained in the incubator for 40 min. Thereafter, cells were loaded with Fura-2/AM in the continuous presence of LiCl. Cells on the first coverslip were exposed to LiCl for 85–90 min prior to stimulation with LTC_4_. The last coverslip in the set had been exposed to LiCl for ∼110 min before stimulation.

##### Knockdown and cDNA Transfection Experiments

For siRNA and cDNA transfection, the Amaxa electroporation system was used (23) with the Amaxa cell line nucleofactor kit T. Transfection efficiency, judged by GFP fluorescence following transfection with a GFP plasmid, was typically ∼50–60%.

STIM1 siRNA was purchased from Life Technologies (catalog no. 4390815). Orai1 siRNA was bought from Origene Technologies (catalog no. SR508429), and the sequences (5′ to 3′) were AGUUCUUACCGCUCAAGAGGCAGGC, CCAUAAGACGGACCGACAGUUCCAG, and AGGGCAGAGUGUGGAAGGAAGAGGC. Both STIM1 and Oria1 siRNAs were used at a final concentration of 50 nm.

PIP5K1α and PIP5K1γ siRNAs and scrambled siRNA were purchased from Dharmacon and used at a final concentration of 75 nm. The PIP5K1α sequences (5′ to 3′) were CGGCAAGAACAUACGAAUU, GCAUCCGGCCUGACGAUUA, GCCCAUGAACAGCGAAAACA, and GAAAAUAGGCCAUCGAAGU. The PIP5K1γ sequences (5′ to 3′) were UCUGGAGAGACUACGUAUA, GCUUCUAUGCCGAGCGCUU, GAGAGGAUGUGCAGUACGA, and GGAGGAGCUGCAUGCGGAA.

Talin1 and talin2 siRNAs were from Dharmacon and used at 50 nm. The Talin1 SMARTpool sequences were CGAGAACUAUGCAGGUAUU, CGAAUGACCAAGGGUAUUA, GUUCGUAGAUUAUCAGACA, and GAGAUGAAGAGUCUACUAU. The Talin2 SMARTpool sequences were UGUUAGUACUCAAGGCGAA, CCGCAAUAAGUGUCGAAUU, CCGCAAAGCUCUUGGCCGA, and GCUAGAAGCAGGUCGGACA.

##### Immunofluorescence

Cells were fixed using 4% paraformaldehyde and then permeabilized with 0.5% Triton X-100. Cells were then blocked using SuperBlock blocking buffer for 1 h at room temperature. Primary antibody (1:200) in PBS was added to the cells and left overnight at 4 °C. Cells were then washed at 10-min intervals in PBS with 0.1% Tween 20 (PBS-T) buffer. Alexa Fluor 488 anti-mouse or anti-rabbit or Alexa Fluor 568 anti-rabbit conjugated antibody was then added for 1 h at room temperature. Cells were mounted onto microscope slides using Vectashield antifade mounting medium with DAPI. Images were obtained using the inverted Olympus FV1000 confocal system equipped with a motorized stage, using a ×60 oil objective of 1.3 numerical aperture and excitation at 488 or 568 nm. All images were grouped according to an image size of 640 × 480 and a step size of 4 μm along the *z* axis. Fluorescence intensities were analyzed for each treatment and normalized to the maximum measured fluorescence using ImageJ.

##### Reverse Transcriptase Polymerase Chain Reaction

QIAshredder was used for homogenization of cell lysate, and total RNA was extracted using the Qiagen RNeasy mini kit. The process of reverse transcription of 1 μg of RNA was achieved using an iScript cDNA synthesis kit. The produced cDNA was amplified utilizing GoTaq Green master mix. The product of the polymerase chain reaction was separated by electrophoresis on 2% agarose gel. Primers were synthesized by Sigma: Talin-1, 5′-TCGGAAGTGGCTTGTGTAGT-3′ (sense) and 5′-GAGAACGCCCGAACTAAACG-3′ (antisense); Talin-2, 5′-GTGGCAGCTAGAGAAACAGC-3′ (sense) and 5′-GGCTTCTTGGATGAGCATGG-3′ (antisense).

##### Western Blot Analysis

Cells were lysed in radioimmunoprecipitation assay lysis buffer supplemented with protease inhibitor mixture, 0.1% Triton X-100, 10 mm sodium metavanadate, and 1 mm PMSF. 15 μg of protein was loaded into 10% SDS-PAGE gel. The protein was next transferred into a nitrocellulose membrane using a semidry protein transfer apparatus (Bio-Rad). 5% nonfat dry milk in phosphate-buffered saline was used to block the membrane. The blocked membrane was then incubated with either PIP5 kinase α or γ antibody (1:2000) for 2 h at room temperature. After washing, a secondary antibody (1:4000) was applied in 5% nonfat dry milk in phosphate-buffered saline solution for 2 h at room temperature. Visualization was accomplished by the use of enhanced chemiluminescence plus the Western blotting detection system. The relative band intensities were analyzed using ImageJ. All bands were normalized to the corresponding β-actin levels as a loading control.

##### Statistical Analysis

All experiments were performed on three independent occasions unless specified otherwise in the text. Independent sample groups were first assessed for normality and equality of variances. To compare single-group treatment, an unpaired Student's *t* test or Mann-Whitney *U* test was used (StatsDirect v2.6.2, Sale, UK). Differences were considered significant at *p* < 0.05. All data are presented in the text and figures as mean ± S.E. (*, *p* < 0.05; **, *p* < 0.01; ***, *p* < 0.001; ****, *p* < 0.0001).

## Results

### 

#### 

##### Oscillations in [Ca^2+^]_i_ Are Sustained by Ca^2+^ Entry through CRAC Channels in RBL-2H3 Cells

**FIGURE 1.**
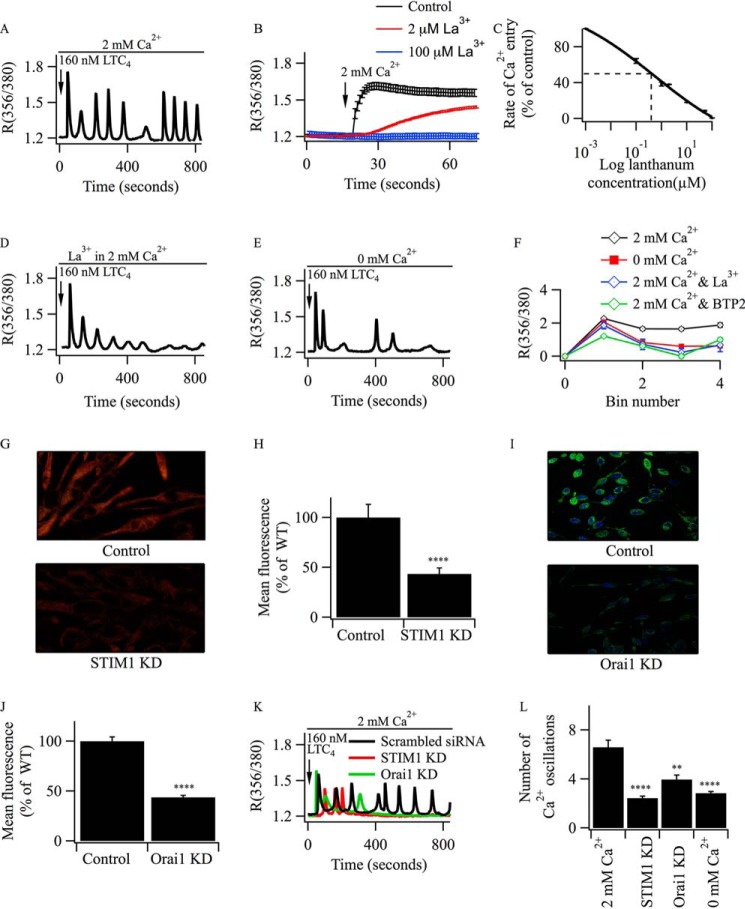
**CRAC channels maintain cytoplasmic Ca^2+^ oscillations in RBL-2H3 cells following leukotriene receptor stimulation.** *A*, typical oscillatory response in [Ca^2+^]*_i_* to 160 nm LTC_4_ in the presence of external calcium. *B*, CRAC channel-mediated Ca^2+^ entry was blocked in a dose-dependent manner by pretreatment with La^3+^. Ca^2+^ influx was evoked by readmission of external Ca^2+^ to cells challenged with 2 μm thapsigargin in Ca^2+^-free solution. Only the Ca^2+^ influx component is shown. The *x* axis represents the time when Ca^2+^ release had returned to resting levels. La^3+^ was applied 3 min prior to thapsigargin exposure. *C*, graph summarizing the La^3+^ dose-inhibition curve. Each point is the mean of between 21 and 32 cells. *D*, oscillations in [Ca^2+^]*_i_* evoked by LTC_4_ in external Ca^2+^ run down in the presence of 10 μm La^3+^ (pretreatment for 3 min). *E*, typical response to LTC_4_ in the absence of external Ca^2+^. *F*, aggregate data (from >50 cells for each treatment) are shown from three independent experiments. Each bin represents a consecutive recording period of 200 s. For most averages, *error bars* (mean ± S.E.) are contained within the symbols. *G*, immunohistochemical staining of STIM1 in either control cells or in cells after knockdown of STIM1. An experiment with a higher than usual transfection efficiency is shown here and in *I. KD*, knockdown. *H*, semiquantitative measurements of mean florescence from two independent experiments for STIM1. ****, *p* < 0.0001. *I*, as in *G*, but Orai1 was measured instead. *J*, histogram showing aggregate data from three independent experiments (each *column* is the average of >50 cells). ****, *p* < 0.0001. *K*, cytoplasmic Ca^2+^ signals in cells stimulated with LTC_4_ after knockdown of STIM1 or Orai1. A scrambled siRNA control is included. *L*, data from three independent experiments are compared. Cells were stimulated with 160 nm LTC_4_. **, *p* < 0.01; ****, *p* < 0.0001.

Activation of cysteinyl leukotriene type I receptors with LTC_4_ in RBL-1 cells evokes a series of oscillations in [Ca^2+^]*_i_* that are supported by Ca^2+^ entry through CRAC channels (9). We repeated these experiments in the RBL-2H3 cell line, which has a stronger secretory phenotype. Stimulation with LTC_4_ resulted in numerous oscillations in [Ca^2+^]*_i_* that were sustained for the duration of the experiment (800 s, Fig. 1*A*), although the amplitude decreased gradually with time because of receptor desensitization (24). To see whether Ca^2+^ entry through CRAC channels was required to maintain the oscillatory response, we used both pharmacological and siRNA-based approaches. La^3+^ is an effective CRAC channel blocker (25) and inhibited the rate of thapsigargin-induced Ca^2+^ influx with an IC_50_ of ∼300 nm (Fig. 1, *B* and *C*). Pre-exposure to 10 μm La^3+^ accelerated the rundown of the oscillations in [Ca^2+^]*_i_* induced by LTC_4_ in 2 mm external Ca^2+^-containing solution (Fig. 1*D*). The rate of rundown in La^3+^ was similar to that seen in cells challenged with LTC_4_ in the absence of external Ca^2+^ (Fig. 1*E*; aggregate data from several experiments are summarized in Fig. 1*F*). Similar results were obtained when 20 μm BTP2 was used to block CRAC channels instead (Fig. 1*F*).

To extend the pharmacological results, we knocked down either STIM1 or Orai1 using siRNA-based approaches. Although both proteins were robustly expressed in wild-type RBL-2H3 cells, knockdown significantly reduced expression (STIM1, Fig. 1, *G* and *H*; Orai1, Fig. 1, *I* and *J*). Oscillations in [Ca^2+^]*_i_* evoked by LTC_4_ in the presence of external Ca^2+^ ran down rapidly after knockdown of either STIM1 or Orai1 (Fig. 1*K*), and the responses resembled those obtained when agonist was applied in the absence of external Ca^2+^ (Fig. 1*L*).

##### Oscillations in [Ca^2+^]_i_ in Response to Leukotriene Receptor Stimulation Run Down in the Presence of Li^+^

**FIGURE 2.**
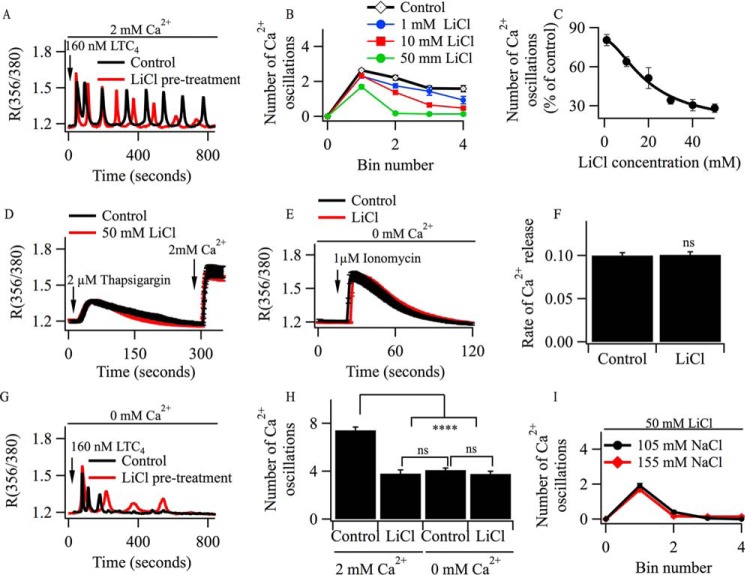
**Li^+^ blocks agonist-mediated oscillations in [Ca^2+^]*_i_*.** *A*, cytoplasmic Ca^2+^ oscillations evoked by 160 nm LTC_4_ are compared between a control cell and one pre-exposed to 10 mm LiCl for ∼90 min. *B*, graph comparing cytoplasmic Ca^2+^ oscillations evoked by LTC_4_ between control cells and cells exposed to different concentrations of LiCl prior to stimulation. *C*, graph plotting the number of cytoplasmic Ca^2+^ oscillations evoked by LTC_4_ over 800 s of stimulation in the presence of different LiCl concentrations. The number of oscillations is normalized to the number of oscillations observed in control cells. Values were fitted with a Hill-type equation, yielding an IC_50_ value of 15 mm. *D*, thapsigargin-evoked Ca^2+^ release and Ca^2+^ influx are compared between control cells and cells pre-exposed to 50 mm LiCl for 90 min. *Error bars* (mean ± S.E.) are within the symbols. *E*, comparison of ionomycin-induced Ca^2+^ release in control cells and cells pretreated with 15 mm of LiCl for 90 min. *F*, data from three independent experiments as in *E. ns*, not significant. *G*, cytoplasmic Ca^2+^ oscillations in Ca^2+^-free external solution following stimulation with 160 nm LTC_4_ are compared between a control cell and one pretreated with 15 mm LiCl for 90 min. *H*, aggregate data from three experiments measuring the total number of cytoplasmic Ca^2+^ oscillations over an 800-s stimulation period with LTC_4_ are compared. Cells were stimulated either in the presence or absence of external Ca^2+^. The LiCl groups refer to pretreatment with 15 mm LiCl for 90 min. The data represent >52 cells from four independent experiments for each condition. ****, *p* < 0.0001. *I*, aggregate data showing the number of cytoplasmic Ca^2+^ oscillations evoked by LTC_4_ in 50 mm LiCl in an external solution containing either 155 or 105 mm NaCl, both with 2 mm Ca^2+^. Each bin number represents a period of 200 s. Each data point is >25 cells.

Li^+^ is an effective inhibitor of inositol monophosphatases and, therefore, should lead to gradual depletion of PIP_2_ levels (18). Preincubation with 10 mm LiCl accelerated the rundown of oscillations in [Ca^2+^]*_i_* evoked by LTC_4_ in the presence of external Ca^2+^ (Fig. 2*A*). The effect of LiCl was concentration-dependent (Fig. 2*B*), and the dose-inhibition curve yielded an IC_50_ of 15 mm (Fig. 2*C*), close to the value reported for IMPase2 (19).

The extent of Ca^2+^ release from the stores evoked by stimulation with either thapsigargin (Fig. 2*D*) or ionomycin (Fig. 2, *E* and *F*) in Ca^2+^-free external solution was unaffected by pretreatment with even high concentrations (50 mm) of Li^+^. Store-operated Ca^2+^ entry, induced by readmission of external Ca^2+^ to cells treated with thapsigargin, was also unaffected by Li^+^ (Fig. 2*D*). Pretreatment with Li^+^ also had no inhibitory effect on the transient oscillatory cytoplasmic Ca^2+^ signals evoked by LTC_4_ when agonist was applied in Ca^2+^-free solution (Fig. 2*G*, aggregate data are summarized in Fig. 2*H*). Collectively, these results rule out an action of Li^+^ on store Ca^2+^ content, store-operated Ca^2+^ entry, and agonist-induced InsP_3_ production.

The time course of rundown of cytoplasmic Ca^2+^ oscillations by LiCl in our experiments is broadly similar to the decline in InsP_3_ levels reported in Chinese hamster ovary cells following muscarinic receptor stimulation (26, 27). In these latter studies, pretreatment with 5–10 mm LiCl for 30 min had no effect on the initial rise in InsP_3_ levels but suppressed the sustained increase that was manifested several minutes after stimulation. We observed no change in the number of oscillations in [Ca^2+^]*_i_* evoked by LTC_4_ in Ca^2+^-free solution (Fig. 2, *G* and *H*), in agreement with these earlier reports showing little effect of LiCl on the initial increase in InsP_3_ levels.

To test for an osmotic effect of LiCl on oscillatory Ca^2+^ signals, we reduced external Na^+^ from 155 to 105 mm and then added 50 mm LiCl. The oscillations in [Ca^2+^]*_i_* evoked by LTC_4_ still ran down quickly (Fig. 2*I*). Therefore, changes in osmolarity are unlikely to explain the accelerated rundown of cytoplasmic Ca^2+^ oscillations in the presence of Li^+^.

##### Inositol Rescues Oscillations in [Ca^2+^]_i_ in Li^+^-treated Cells but Only in the Presence of Ca^2+^ Entry through CRAC Channels

**FIGURE 3.**
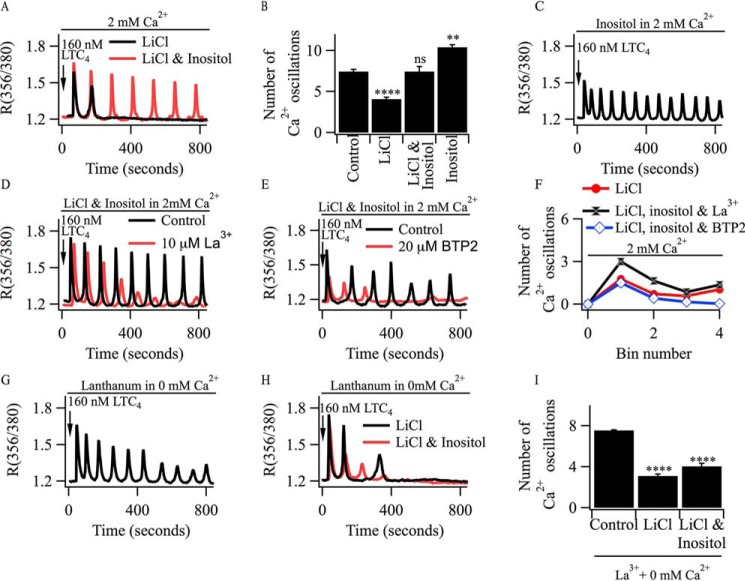
**Inositol rescues cytoplasmic Ca^2+^ oscillations in LiCl-treated cells.** *A*, oscillations in cytoplasmic [Ca^2+^]*_i_* evoked by LTC_4_ are sustained in a LiCl-treated cell when 10 mm inositol is added shortly before stimulation. *B*, aggregate data comparing the number of oscillations over an 800-s recording period for the conditions shown. Each *column* is the average of >30 cells from four independent experiments. **, *p* < 0.01; ****, *p* < 0.0001; *ns*, not significant. *C*, exposure to 10 mm inositol increases the oscillation frequency in a control cell. *D* and *E*, inositol fails to rescue oscillations in [Ca^2+^]*_i_* evoked by LTC_4_ in a LiCl-treated cell when CRAC channels are blocked with either La^3+^ (*D*) or BTP2 (*E*). *F*, aggregate data are compared for >50 cells/point. Each bin number reflects 200 s of recording. *G*, cytoplasmic Ca^2+^ oscillations are maintained over 800 s when a cell is challenged with LTC_4_ in Ca^2+^-free solution containing 1 mm La^3+^. *H*, Ca^2+^ oscillations run down in 0 Ca^2+^solution containing 1 mm La^3+^ following pretreatment with 15 mm LiCl, and this cannot be rescued by inositol. *I*, aggregate data are compared. Cells were stimulated with LTC_4_ in 0Ca^2+^ solution containing 1 mm La^3+^. ****, *p* < 0.0001.

In the blowfly salivary gland, a seminal study by Fain and Berridge (28) has demonstrated that application of inositol rescued serotonin receptor-induced Ca^2+^ flux that had subsided in the continuous presence of agonist. We therefore examined whether the presence of exogenous inositol could rescue the cytoplasmic Ca^2+^ oscillations in Li^+^-treated cells. Although oscillations in [Ca^2+^]*_i_* evoked by LTC_4_ ran down in the presence of LiCl (Fig. 3, *A* and *B*), pre-exposure to 10 mm inositol for a few minutes prior to stimulation rescued the oscillations in [Ca^2+^]*_i_* (Fig. 3*A*; aggregate data are shown in Fig. 3*B* and compared with the control response). Interestingly, addition of inositol to control cells prior to stimulation with LTC_4_ in the presence of external Ca^2+^ resulted in an increase in the frequency of oscillations in [Ca^2+^]*_i_* after agonist was added (Fig. 3, *B* and *C*).

Although inositol rescued oscillations in [Ca^2+^]*_i_* in Li^+^-treated cells, Ca^2+^ entry through CRAC channels was still required to sustain the oscillatory response. Exposure to the CRAC channel blocker La^3+^ accelerated the rundown of cytoplasmic Ca^2+^ oscillations in Li^+^-treated cells despite the presence of inositol (Fig. 3*D*; aggregate data are shown in Fig. 3*F*). Similar results were obtained when BTP2 was used to block the CRAC channels instead (Fig. 3, *E* and *F*).

The rundown of oscillations in [Ca^2+^]*_i_* in Li^+^-treated cells when CRAC channels are blocked, despite the presence of inositol, could reflect a role for Ca^2+^ entry in the synthesis of PIP_2_ from inositol. Alternatively, loss of the oscillatory response might simply arise from compromised store refilling. To distinguish between these possibilities, we took advantage of a protocol that enables store refilling to occur but in the absence of Ca^2+^ entry through CRAC channels. Cytoplasmic Ca^2+^ oscillations can be sustained in the absence of external Ca^2+^ when Ca^2+^ extrusion across the plasma membrane is inhibited, for example with high (millimolar) concentrations of La^3+^ (9, 10). Under these conditions, Ca^2+^ released from the stores cannot be exported out of the cell and so is taken back into the ER, thereby maintaining store Ca^2+^ content in readiness for the next oscillatory cycle in [Ca^2+^]*_i_*. Stimulation with LTC_4_ in Ca^2+^-free solution supplemented with 1 mm La^3+^ resulted in the generation of numerous oscillations in [Ca^2+^]*_i_* (Fig. 3*G*). Preincubation with LiCl led to rundown of the cytoplasmic Ca^2+^ oscillations (Fig. 3*H*). However, inositol no longer rescued the oscillatory response in Li^+^-treated cells (Fig. 3*H*; aggregate data are shown in Fig. 3*I*). These results, therefore, reveal a major role for Ca^2+^ influx through CRAC channels in replenishment of the PIP_2_ pool from inositol.

##### Inhibition of Phosphatidylinositol 4 Kinase Accelerates the Rundown of Agonist-driven Oscillations in [Ca^2+^]_i_

**FIGURE 4.**
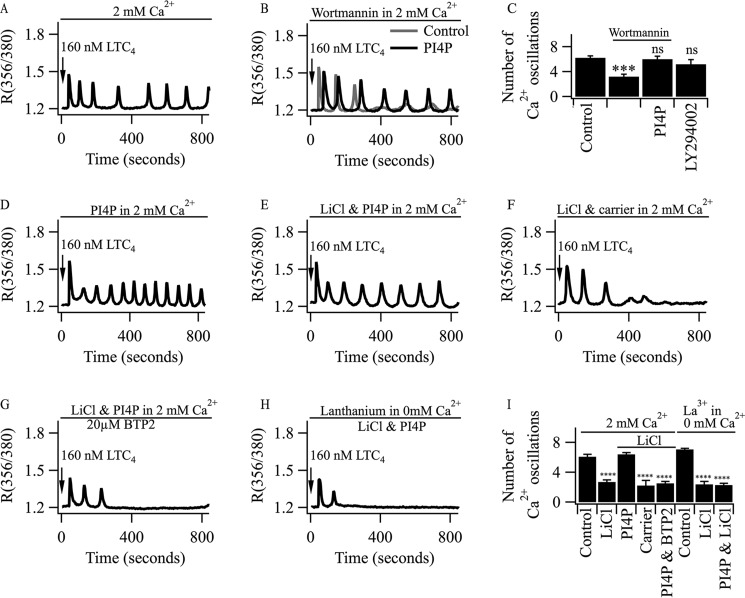
**Involvement of PI4P in supporting oscillations in [Ca^2+^]*_i_* evoked by LTC_4_.** *A*, a typical control recording showing oscillations in [Ca^2+^]*_i_* evoked by LTC_4_ in the presence of external Ca^2+^. *B*, wortmannin (10 μm, 10-min pretreatment) accelerates rundown of the cytoplasmic Ca^2+^ oscillations. Rundown is less pronounced when PI4P (70 μm, 7-min pretreatment) is applied prior to stimulation. *C*, aggregate data from three independent experiments are compared. Each *column* represents data from >30 cells. ***, *p* < 0.001; *ns*, not significant. *D*, pretreatment with PI4P increases the cytoplasmic Ca^2+^ oscillation frequency. *E*, pretreatment with PI4P rescues oscillations in [Ca^2+^]*_i_* in a cell pre-exposed to LiCl for 105 min. *F*, cytoplasmic Ca^2+^ oscillations evoked by LTC_4_ are not rescued in LiCl-treated cells when the carrier for PI4P is used instead. *G*, PI4P fails to rescue oscillations in [Ca^2+^]*_i_* in Li^+^-treated cells in the presence of BTP2. *H*, PI4P has no effect on the rundown of cytoplasmic Ca^2+^ oscillations in Ca^2+^-free solution containing 1 mm La^3+^ in a LiCl-treated cell. *I*, aggregate data comparing the number of Ca^2+^ oscillations over an 800-s recording period for the different conditions are shown. La^3+^ in 0 mm Ca^2+^ denotes 0 Ca^2+^ supplemented with 1 mm La^3+^. ****, *p* < 0.0001.

The preceding results suggest that Ca^2+^ influx through CRAC channels helps replenish PIP_2_ levels during cytoplasmic Ca^2+^ oscillations. This would mean that significant amounts of PIP_2_ are hydrolyzed following stimulation with LTC_4_ and that, therefore, phosphatidylinositol 4- and 5-kinases should be active during stimulation. A simple prediction is therefore that inhibition of these kinases should accelerate the rundown of the oscillations in [Ca^2+^]*_i_* because of the loss of PIP_2_ production. At relatively high concentrations, wortmannin inhibits phosphatidylinositol 4- kinase (29, 30) and, therefore, impairs PIP_2_ resynthesis. Compared with control responses, pretreatment with 10 μm wortmannin resulted in faster rundown of cytoplasmic Ca^2+^ oscillations evoked by LTC_4_ (compare the control response in Fig. 4*A* with that evoked in the presence of wortmannin in Fig. 4*B*). The number of Ca^2+^ oscillations that were triggered by LTC_4_ in the presence of wortmannin (Fig. 4*C*) was similar to the number seen in Ca^2+^-free solution (Fig. 1*L*). A previous study reported that wortmannin inhibited store-operated Ca^2+^ entry through inhibition of phosphatidylinositol 4-kinase and, thereby, depletion of PIP_2_ (31). In that report, a prominent inhibition of Ca^2+^ influx was seen when wortmannin pretreatment was combined with exposure to the agonist methacholine to ensure PIP_2_ depletion. Pretreatment with wortmannin (20 μm) but in the absence of methacholine had no inhibitory effect on Ca^2+^ entry (31). These data are consistent with our previous report showing that wortmannin alone had no effect on CRAC channel activity in RBL cells (32). Furthermore, loss of cytoplasmic Ca^2+^ oscillations evoked by LTC_4_ in the presence of wortmannin could be rescued by exogenous PI4P (Fig. 4, *B* and *C*), consistent with an action by wortmannin on phosphatidylinositol 4-kinase. Wortmannin also blocks phosphatidyl inositol 3-kinase. However, pretreatment with the PI 3-kinase inhibitor LY294002 did not mimic the inhibitory effect of wortmannin on oscillations evoked by LTC_4_ (Fig. 4*C*).

##### Application of Phosphatidylinositol 4-Phosphate Rescues Oscillations in [Ca^2+^]_i_ Evoked by LTC_4_ in Li^+^-treated Cells

The results described above with wortmannin suggest that PI4P production is important for sustaining oscillations in [Ca^2+^]*_i_* evoked by LTC_4_. To test this more directly, we applied purified PI4P to Li^+^-treated cells to see whether this could prevent rundown of the cytoplasmic Ca^2+^ oscillations. In control cells not treated with LiCl, exposure to PI4P led to a small increase in the number of oscillations in [Ca^2+^]*_i_* induced by LTC_4_ (Fig. 4*D*). Pre-exposure to PI4P prevented the rundown of the oscillations in [Ca^2+^]*_i_* in Li^+^-treated cells (Fig. 4*E*). By contrast, the histone carrier for PI4P failed to rescue the oscillations in [Ca^2+^]*_i_* in Li^+^-treated cells (Fig. 4*F*). Blocking of CRAC channels with BTP2 prevented the rescue of the cytoplasmic Ca^2+^ oscillations by PI4P in Li^+^-treated cells (Fig. 4*G*), demonstrating that Ca^2+^ influx was still important for maintaining the response despite the presence of PI4P. Aggregate data for the various conditions are summarized in Fig. 4*I*. Importantly, as was the case with inositol (Fig. 3*H*), PI4P failed to rescue cytoplasmic Ca^2+^ oscillations evoked by LTC_4_ when agonist was applied to Li^+^-treated cells in Ca^2+^-free external solution containing 1 mm La^3+^ (Fig. 4, *H* and *I*). Rescue of oscillations in [Ca^2+^]*_i_* in Li^+^-treated cells by PI4P therefore requires Ca^2+^ influx and places the Ca^2+^-dependent site downstream of PI4P production.

##### Knockdown of PIP5 Kinase I Abolishes Oscillations in [Ca^2+^]_i_ Evoked by LTC_4_

**FIGURE 5.**
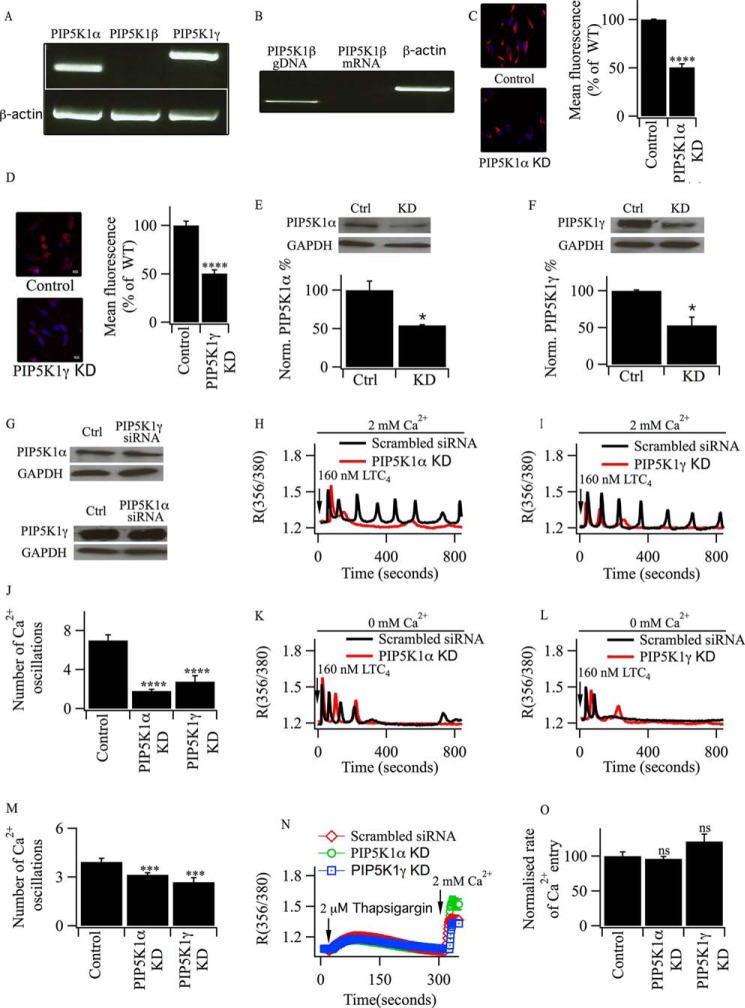
**PIP5K1 isoforms are involved in maintaining LTC_4_-driven cytoplasmic Ca^2+^ oscillations.** *A*, RT-PCR comparing the expression of PIP5K1 isoforms in RBL-2H3 cells. *B*, PIP5KIβ mRNA is absent from RBL-2H3 cells despite genomic DNA (*gDNA*) being detectable. *C* and *D*, confocal microscopy images comparing the expression of PIPK1α and γ protein between control cells and those in which either PIP5K1α (*C*) or PIP5K1γ (*D*) had been knocked down (*KD*). The corresponding histograms summarize data from >40 cells in each group. ****, *p* < 0.0001. *E*, Western blot comparing the expression of PIP5K1α in control (*Ctrl*) cells and after siRNA-directed knockdown. *, *p* < 0.05. *F*, Western blot comparing the expression of PIP5K1γ in control cells and after siRNA-directed knockdown. *, *p* < 0.05. *G*, Western blot comparing the expression of PIP5K1α after knockdown of PIP5Kγ (*top panel*) and vice versa (*bottom panel*). *H* and *I*, cytoplasmic Ca^2+^ oscillations evoked by LTC_4_ run down quickly after knockdown of PIP5K1α (*H*) or PIP5K1γ (*I*). Scrambled siRNA controls are included. *J*, histogram comparing the total number of cytoplasmic Ca^2+^ oscillations evoked by LTC_4_ in 2 mm external Ca^2+^ over 800 s of stimulation from three independent experiments. ****, *p* < 0.0001. *K* and *L*, the effect of knockdown of PIP5K1α (*K*) or PIP5K1γ (*L*) on responses evoked by LTC_4_ in Ca^2+^-free solution. *M*, histogram comparing the average number of Ca^2+^ oscillations in Ca^2+^-free solution for the conditions shown. ***, *p* < 0.001. *N*, Ca^2+^ release and Ca^2+^ influx evoked by thapsigargin are compared following knockdown of PIP5K1α and PIP5K1γ or after transfection with scrambled siRNA. *O*, the rates of Ca^2+^ entry following stimulation with thapsigargin as in *K* are compared for the conditions shown. *ns*, not significant.

PI4P is converted to PIP_2_ by type I PIP kinases, which consist of three isoforms: PIP5 kinase 1α, 1β, and 1γ (33, 34). RT-PCR revealed the presence of PIP5 kinase1α and 1γ in RBL-2H3 cells but not the β isoform (Fig. 5, *A* and *B*). The presence of these proteins was confirmed using immunocytochemistry (Fig. 5, *C* and *D*) and Western blots (Fig. 5, *E* and *F*). SiRNA against either PIP5 kinase 1α or PIP5 kinase 1γ resulted in an ∼60% reduction in protein expression (Fig. 5, *C–F*). Knockdown of PIP5 kinase 1α had no effect on the expression of PIP5 kinase 1γ and *vice versa* (Fig. 5*G*). Following knockdown of PIP5 kinase 1α, the typical oscillatory response in [Ca^2+^]*_i_* evoked by LTC_4_ in the presence of external Ca^2+^ was abolished (Fig. 5, *H* and *J*). Similar results were observed when PIP5 kinase 1γ was knocked down instead (Fig. 5, *I* and *J*), suggesting redundancy between these isoforms. Knockdown of either PIP5 kinase isoform had only a modest effect on responses in Ca^2+^-free solution (Fig. 5, *K–M*). Neither the store Ca^2+^ content, as measured by the response to thapsigargin in Ca^2+^-free solution, nor the rate of store-operated Ca^2+^ influx were affected by knockdown of either PIP5 kinase isoform (Fig. 5, *N* and *O*).

**FIGURE 6.**
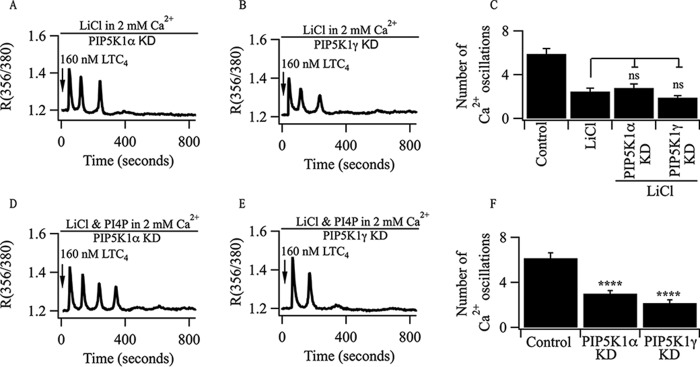
**PI4P fails to rescue oscillations in [Ca^2+^]*_i_* evoked by LTC_4_ following knockdown of PIP5K1α or PIP5K1γ in LiCl-treated cells.** *A* and *B*, typical transient oscillations in [Ca^2+^]*_i_* evoked by LTC_4_ following knockdown (*KD*) of either PIP5K1α (*A*) or PIP5K1γ (*B*). *C*, aggregate data are summarized. *ns*, not significant. *D* and *E*, PI4P (70 μm) fails to rescue the cytoplasmic Ca^2+^ oscillations evoked by LTC_4_ in either PIP5K1α- (*D*) or PIP5K1γ-deficient (*E*) cells pre-treated with LiCl. *F*, aggregate data for the conditions shown are summarized. Each *column* represents results from >19 cells. ****, *p* < 0.0001.

Oscillations in [Ca^2+^]*_i_* in Li^+^-treated cells challenged with LTC_4_ in the presence of external Ca^2+^ run down within a few minutes (Fig. 2, *A* and *B*) but can be rescued by PI4P (Fig. 4, *E* and *I*). To see whether this rescue required conversion of PI4P to PIP_2_, we knocked down either PIP5 kinase 1α or 1γ and examined whether this prevented PI4P from rescuing the cytoplasmic Ca^2+^ oscillations evoked by LTC_4_. In Li^+^-treated cells in which PIP5 kinase 1α or 1γ had been knocked down, oscillations in [Ca^2+^]*_i_* ran down within 5 min (Fig. 6, *A–C*). However, under these conditions, application of PI4P no longer rescued the cytoplasmic Ca^2+^ oscillations (Fig. 6, *D–F*).

##### Prolonged Agonist-evoked Oscillations in [Ca^2+^]_i_ Require Talin1

**FIGURE 7.**
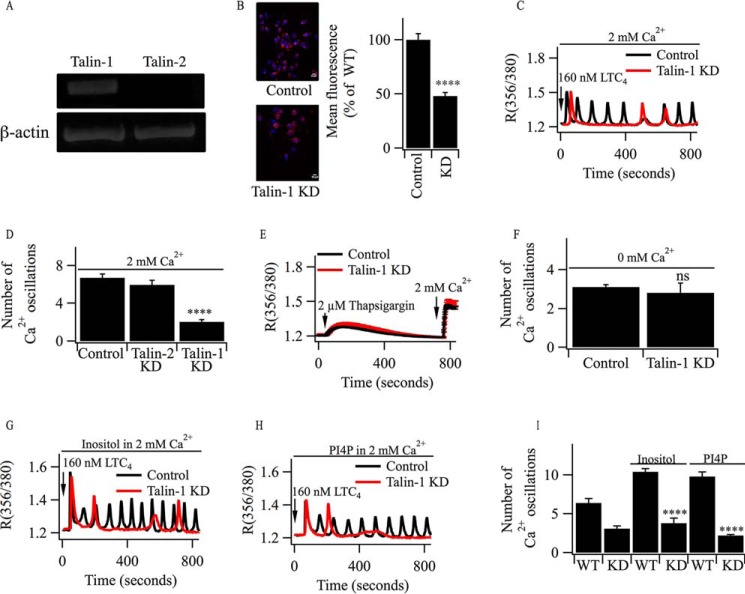
**Knockdown of talin1 accelerates the rundown of oscillations in [Ca^2+^]*_i_* evoked by LTC_4_.** *A*, RT-PCR reveals the presence of talin1 but not talin2 in RBL-2H3 cells. *B*, immunocytochemical detection of talin1 in control cells and after knockdown (*KD*). The histogram summarizes data from between 40 and 52 cells for each condition. ****, *p* < 0.0001. *C*, cytoplasmic Ca^2+^ oscillations evoked by LTC_4_ run down more quickly after talin1 knockdown. *D*, aggregate data from several experiments are summarized. Each *column* represents data from between 24 and 32 cells. *E*, thapsigargin-evoked cytoplasmic Ca^2+^ signals are unaffected by talin1 knockdown. *F*, the total number of cytoplasmic Ca^2+^ oscillations evoked by LTC_4_ in Ca^2+^-free solution are similar in control cells and those in which talin1 had been knocked down. *ns*, not significant. *G*, oscillations in [Ca^2+^]*_i_* evoked by LTC_4_ in talin1-deficient cells are not rescued by addition of inositol. *Control* refers to a wild-type cell stimulated with LTC_4_ in the presence of inositol. *H*, PI4P fails to rescue cytoplasmic Ca^2+^ oscillations in cells following knockdown of talin1. *I*, aggregate data from several independent experiments are compared. ****, *p* < 0.0001 compared with the corresponding controls. LTC_4_ was used to stimulate the cells. *KD*, knockdown of talin1.

To identify a potential mechanism linking Ca^2+^ influx to PIP_2_ replenishment, we sought Ca^2+^-dependent proteins that regulate PIP5 kinase activity. One candidate is talin, an adaptor protein that links integrin to the actin cytoskeleton (35). There are two homologous talin isoforms, talin1 and talin2. The NH_2_-terminal globular head has a band4.1/ezrin/radixin/moesin-like domain that binds actin, PIP_2_, and PIP5 kinase (35, 36). Talins can be cleaved by the Ca^2+^-dependent protease calpain (37), providing a possible link between cytoplasmic Ca^2+^ and PIP5 kinase activity. Talin1 mRNA was expressed in RBL-2H3 cells, whereas talin2 was undetectable (Fig. 7*A*). Immunocytochemical studies confirmed the presence of talin1, and this was reduced by ∼50% following knockdown (Fig. 7*B*). Oscillations in [Ca^2+^]*_i_* evoked by LTC_4_ ran down considerably more quickly following talin1 knockdown (Fig. 7, *C* and *D*). Neither store-operated Ca^2+^ influx, measured by stimulating cells with thapsigargin (Fig. 7*E*), nor Ca^2+^ release in the absence of Ca^2+^ influx (Fig. 7*F*) were impaired following talin1 knockdown. Pre-exposure to either inositol (Fig. 7*G*) or PI4P (Fig. 7*H*) did not rescue cytoplasmic Ca^2+^ oscillations to LTC_4_ following knockdown of talin1. Aggregate data from several such experiments are summarized in Fig. 7*I*.

##### Independence of P2Y and cysLT1 Receptors

CysLT1 receptor-dependent Ca^2+^ signaling is affected by the presence of caveolin-1 and is disrupted by removal of cholesterol from the plasma membrane by methyl-β-cyclodextrin (MβCD), suggesting the leukotriene receptors signal within lipid raft domains (23).

**FIGURE 8.**
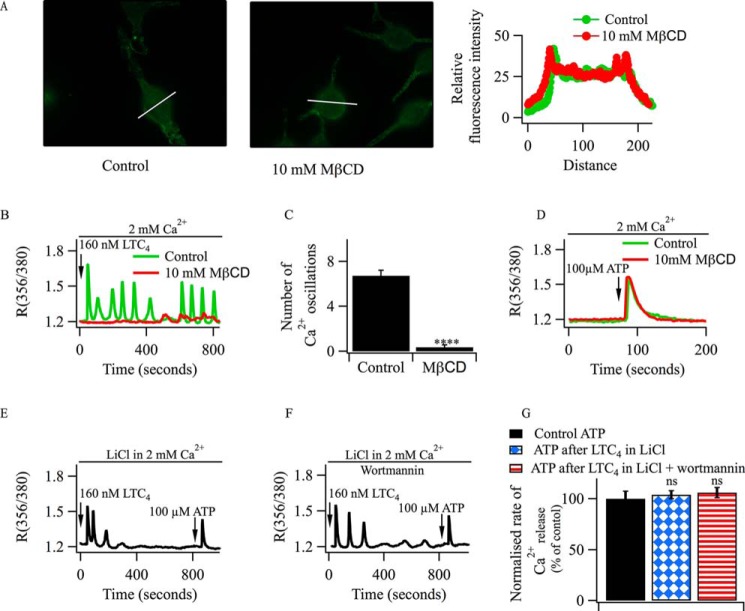
**Non-overlap of PIP_2_ pools in RBL-2H3 cells.** *A*, confocal microscopic images compare the distribution of FLAG-tagged CysLT1 receptors following stimulation with 160 nm LTC_4_ in control cells and in cells pretreated with 10 mm MβCD for 30 min. The fluorescence profiles from the line scans are shown in the corresponding graph. *B*, the typical oscillatory Ca^2+^ signal induced by LTC_4_ in a control cell is lost following treatment with MβCD. *C*, aggregate data analyzing the number of oscillations from three independent experiments (34 cells for each condition). ****, *p* < 0.0001. *D*, the Ca^2+^ response induced by ATP is unaffected by the presence of MβCD (10 mm, 30 min pretreatment). *E*, after cytoplasmic Ca^2+^ oscillations evoked by LTC_4_ had run down in a cell pretreated with 15 mm LiCl for 90 min, application of ATP elicited prominent Ca^2+^ release. *F*, ATP application evoked Ca^2+^ release after the oscillatory response to LTC_4_ had run down in the presence of 10 μm wortmannin and LiCl. *G*, aggregate data from three independent experiments (between 26 and 30 cells/column) are compared. When LiCl (15 mm) was present, it was preincubated for 90 min prior to challenge with LTC_4_. *ns*, not significant.

In RBL-1 cells, stimulation of P2Y receptors with ATP generates InsP_3_, which then triggers Ca^2+^ release from the stores. P2Y-dependent responses are unaffected by MβCD, indicating that they are not located close to cysLT1 receptors (23). To test whether P2Y and cysLT1 receptors coupled to the same pool of PIP_2_, we designed experiments to see whether ATP was able to evoke InsP_3_-dependent Ca^2+^ release after responses to LTC_4_ had ceased. We first confirmed that previous findings made in RBL-1 cells also occurred in the RBL-2H3 cell line. Immunocytochemical studies showed expression of cysLT1 receptors in the cell periphery, and this was not altered by MβCD (Fig. 8*A*). Oscillations in [Ca^2+^]*_i_* evoked by LTC_4_ were abolished by MβCD (Fig. 8, *B* and *C*). However, ATP still elicited a large Ca^2+^ transient in the presence of MβCD (Fig. 8*D*).

After cytoplasmic Ca^2+^ oscillations evoked by LTC_4_ had run down in Li^+^-treated cells, we applied ATP to see whether Ca^2+^ release could still be evoked. A robust Ca^2+^ transient occurred (Fig. 8, *E* and *G*), revealing that P2Y receptors were able to couple to phospholipase C under conditions where cysLT1 receptors could not.

To ensure that there was no PIP_2_ resynthesis during the interval between LTC_4_ exposure and ATP stimulation, we pretreated cells with both LiCl and wortmannin. After oscillations in [Ca^2+^]*_i_* following challenge with LTC_4_ had run down (Fig. 8*F*), subsequent exposure to ATP elicited robust Ca^2+^ release (Fig. 8, *F* and *G*).

## Discussion

**FIGURE 9.**
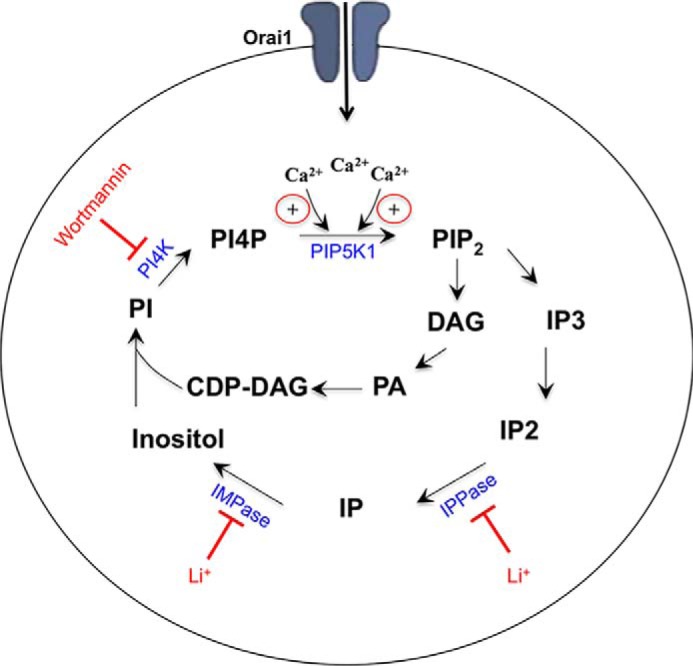
**Schematic summarizing how calcium influx affects the PIP_2_ pathway.** Both inositol polyphosphatase (*IPPase*) and IMPase are targets of Li^+^. *CDP-DAG*, cytidine diphosphate-diacylglycerol; *DAG*, diacylglycerol; *PA*, phosphatidic acid; *IP*, inositol phosphate.

Our findings reveal a role for Ca^2+^ entry through CRAC channels in regulating PIP_2_ replenishment and, therefore, in supporting oscillations in [Ca^2+^]*_i_* following physiological levels of cysLT1 receptor stimulation. A schematic summarizing our main findings and the sites of intervention is shown in Fig. 9.

Cytoplasmic Ca^2+^ oscillations evoked by LTC_4_ ran down in the presence of Li^+^, an inhibitor of inositol monophosphatases (18), demonstrating that PIP_2_ resynthesis was required for maintaining the Ca^2+^ response. Direct application of either inositol or phosphatidylinositol 4-phosphate was able to rescue the oscillatory Ca^2+^ response in the presence of Li^+^, consistent with an action of Li^+^ on inositol monophosphatase. However, recovery of the oscillations in [Ca^2+^]*_i_* evoked by LTC_4_ was prevented by block of CRAC channel activity. Our results suggest that Ca^2+^ influx is required to replenish the PIP_2_ pool that is targeted by cysLT1 receptors. Phosphatidylinositol 4-phosphate is phosphorylated to PIP_2_ by three isoforms of the type I PIP5 kinase (33) that are localized to various membrane compartments, including the plasma membrane, through a dilysine motif and another conserved lysine within the activation loop, a stretch of ∼20 amino acids in the C terminus (38). RBL-2H3 cells expressed both PIP5 kinase α and γ, and knockdown of either accelerated rundown of the cytoplasmic Ca^2+^ oscillations evoked by LTC_4_. The simplest explanation for our results is, therefore, that Ca^2+^ entry through CRAC channels stimulates PIP5 kinase α/γ to convert phosphatidylinositol 4-phosphate to PIP_2_ and, therefore, ensure adequate levels of the phospholipid for sustained cysLT1 receptor-dependent Ca^2+^ signaling. Type I PIP5 kinases are regulated by a variety of signals, including small G-proteins such as Rho, Rac, and Arf; phosphatidic acid; phosphorylation by tyrosine kinases; and the cytoskeletal protein talin 1 (33). Many of these pathways exhibit Ca^2+^ dependence, and it is therefore conceivable that Ca^2+^ entry modulates a regulatory pathway that serves to stimulate PIP5 kinase. Our data using an siRNA-based approach suggest a role for talin1, although how the protein is activated by Ca^2+^ influx and whether it is actively involved in supporting oscillations in [Ca^2+^]*_i_* or has more of a housekeeping role remains unclear.

Ca^2+^ influx might also stimulate PI transfer from the peripheral ER to the plasma membrane. Recent work has shown that extended synaptotagmins are activated by cytoplasmic Ca^2+^ and serve to bring the subplasmalemmal ER closer to the plasma membrane, where phosphatidylinositol transfer proteins, including Nir2, shuttle PI from the ER to the plasma membrane (39). Although such a mechanism might contribute to PIP_2_ replenishment under our conditions, our data suggest that the key step that is regulated by Ca^2+^ influx following cysLT1 receptor activation is the conversion of phosphatidylinositol 4-phosphate to PIP_2_ by PIP5 kinase, a reaction that takes place mainly in the plasma membrane.

The frequency of InsP_3_-dependent cytoplasmic Ca^2+^ oscillations can be altered by varying the rate or extent of Ca^2+^ entry (40). We now find that oscillation frequency evoked by LTC_4_ can also be increased by supply of exogenous inositol or PI4P. This suggests that PIP_2_ availability is rate-limiting. Providing more precursor accelerates its replenishment, and this increases the frequency of oscillations in [Ca^2+^]*_i_*.

Growing evidence points to the existence of local pools of PIP_2_ within the plasma membrane (41). Our functional studies are consistent with this view and suggest that these local pools exchange slowly. Oscillations in [Ca^2+^]*_i_* evoked by cysLT1 receptor activation ran down quickly in the presence of Li^+^ and wortmannin, which inhibit inositol monophosphatases and PI4P kinases, respectively. Nevertheless, subsequent stimulation of P2Y receptors, which also couple to phospholipase Cβ, elicited Ca^2+^ release that was similar in size and kinetics to responses obtained in the absence of the inhibitors. Therefore, P2Y receptors access a PIP_2_ pool that is distinct from that utilized by cysLT1 receptors.

In summary, our findings identify a role for Ca^2+^ entry through CRAC channels in maintaining a plasma membrane pool of PIP_2_ that is accessible to cysLT1 receptors. Ca^2+^ influx, therefore, has two important roles in controlling cytoplasmic Ca^2+^ oscillations: maintaining PIP_2_ levels and refilling the intracellular Ca^2+^ stores. The former will be important in initiating each oscillation in [Ca^2+^]*_i_*, whereas the latter will help set the interspike interval. Both will affect spike frequency but through distinct mechanisms. Stimulation of PIP_2_ production constitutes an interesting autoregulatory mechanism through which CRAC channels will be able to sustain their activity by ensuring that there are sufficient PIP_2_ levels in the plasma membrane to produce InsP_3_ and, therefore, store depletion, which is required for the opening of the channels. How Ca^2+^ influx activates PIP5 kinase and whether this is driven by local Ca^2+^ entry requires further investigation.

## Author Contributions

A. A. and A. B. P. designed the study. A. A. performed and analyzed the experiments. A. B. P. wrote the manuscript. Both authors approved the final version of the manuscript.

## Footnotes

3 The abbreviations used are:

PIP_2_: phosphatidylinositol 4,5-bisphosphate
InsP_3_: inositol trisphosphate
ER: endoplasmic reticulum
IMPase: inositol monophosphatase
PI: phosphatidylinositol
PIP5: phosphatidylinositol 4-phosphate 5
PI4P: phosphatidylinositol 4-phosphate
LTC_4_: leukotriene C4
MβCD: methyl-β-cyclodextrin
CRAC: Ca^2+^ release-activated Ca^2+^
STIM: stromal interaction molecule.
